# Antigen Discovery, Bioinformatics and Biological Characterization of Novel Immunodominant *Babesia microti* Antigens

**DOI:** 10.1038/s41598-020-66273-6

**Published:** 2020-06-12

**Authors:** Nitin Verma, Ankit Puri, Edward Essuman, Richard Skelton, Vivek Anantharaman, Hong Zheng, Siera White, Karthigayan Gunalan, Kazuyo Takeda, Surabhi Bajpai, Timothy J. Lepore, Peter J. Krause, L. Aravind, Sanjai Kumar

**Affiliations:** 1 Laboratory of Emerging Pathogens, Division of Emerging and Transfusion Transmitted Diseases, Office of Blood Research and Review, Center for Biologics Evaluation and Research, Food and Drug Administration, Silver Spring, MD 20993 USA; 2 Laboratory of Malaria and Vector Research, National Institute of Allergy and Infectious Diseases, National Institutes of Health, Rockville, MD 20852 USA; 3 Lab Of Method Development, Office of Vaccine Research and Review, Center for Biologics Evaluation and Research, Food and Drug Administration, Silver Spring, MD 20993 USA; 4 Department of Bioscience and Biotechnology, Banasthali Vidyapith, Banasthali, 304022 India; 5 Yale School of Public Health and Yale School of Medicine, New Haven, CT 06520 USA; 6 National Center for Biotechnology Information, National Library of Medicine, National Institutes of Health, Bethesda, MD 20894 USA; 7 Nantucket Cottage Hospital, Nantucket, MA 02554 USA

**Keywords:** High-throughput screening, Parasite genomics

## Abstract

*Babesia microti* is an intraerythrocytic parasite and the primary causative agent of human babesiosis. It is transmitted by *Ixodes* ticks, transfusion of blood and blood products, organ donation, and perinatally. Despite its global public health impact, limited progress has been made to identify and characterize immunodominant *B. microti* antigens for diagnostic and vaccine use. Using genome-wide immunoscreening, we identified 56 *B. microti* antigens, including some previously uncharacterized antigens. Thirty of the most immunodominant *B. microti* antigens were expressed as recombinant proteins in *E. coli*. Among these, the combined use of two novel antigens and one previously described antigen provided 96% sensitivity and 100% specificity in identifying *B. microti* antibody containing sera in an ELISA. Using extensive computational sequence and bioinformatics analyses and cellular localization studies, we have clarified the domain architectures, potential biological functions, and evolutionary relationships of the most immunodominant *B. microti* antigens. Notably, we found that the BMN-family antigens are not monophyletic as currently annotated, but rather can be categorized into two evolutionary unrelated groups of BMN proteins respectively defined by two structurally distinct classes of extracellular domains. Our studies have enhanced the repertoire of immunodominant *B. microti* antigens, and assigned potential biological function to these antigens, which can be evaluated to develop novel assays and candidate vaccines.

## Introduction

*Babesia microti*, an intraerythrocytic parasite belonging to the phylum Apicomplexa, is the primary causative agent of human babesiosis^1^. It is transmitted by *Ixodes* ticks but also can be transmitted by transfusion of blood, blood products, solid organ donation, and perinatal transmission^2–7^. Human *B. microti* infection occurs worldwide but is most commonly reported in the United States and is endemic in the Northeast and upper Midwest^1^. The number of tick-borne and transfusion-transmitted cases of babesiosis in the United States has risen dramatically in the past two decades^5,8^. New cases of babesiosis have been reported throughout the world, including a new *Babesia* endemic area in Northeast China due to *Babesia venatorum*^9^. Asymptomatic *B. microti* infection is not uncommon in healthy residents of *B. microti*-endemic regions and among blood donors. On Block Island, Rhode Island, about a fifth of healthy adults infected with *B. microti* are asymptomatic^10^. In a comprehensive study in 60,512 healthy blood donors in endemic areas in northeastern United States, 0.38% of the donors were shown to carry *B. microti* DNA or *B. microti* antibody in their bloodstream^11^. Asymptomatically infected individuals may transmit the infection through the blood supply if they donate blood. Death occurs in about a fifth of individuals receiving contaminated blood transfusion^5^. Similar fatality rates have been reported in asplenic patients and in patients suffering from cancer with or without asplenia and/or rituximab therapy. The latter group of patients often require hospital admission and experience persistent relapsing disease that may last for more than a year^11–13^. Fatality estimates for *B. microti* infection have ranged from <1% to 2% in the general population and 3% to 9% in hospitalized babesiosis patients^14–19^. Fatality rates among asplenic European patients with *Babesia divergens* infection have reached as high as 42% but more recently have decreased as a result of improved adjunctive therapy^19–21^.

Although the full genome sequence for *B. microti* was published in 2012^22,23^, there is still a scarcity of well-characterized, immunodominant *B. microti* antigens for use in diagnostic assays and vaccine development. Antibody testing using the Indirect Fluorescence Antibody assay (IFA) has been used in conjunction with *B. microti* PCR as an effective blood donor screening approach to prevent transfusion transmitted babesiosis^11,24^. Earlier efforts using cDNA expression libraries have led to the identification of novel *B. microti* immunodominant antigens which have proven useful as diagnostic markers for infection^25–27^. The availability of full genome sequence for *B. microti*^22,23^ allows the genome-wide screening to identify novel immunodominant *B. microti* antigens and to perform genetic and biological characterization of these antigens. Such investigations may not only contribute to the development of superior diagnostic assays but also provide a better understanding of *Babesia* pathogenesis and phylogenetic relationships among different *Babesia* species/strains.

In this work, we generated *B. microti* cDNA phage display libraries to identify a battery of novel immuno-dominant *B. microti* antigens, searched for structural features that could predict their biological function and cellular localization, and corrected the annotation and phylogenetic classification of some previously discovered antigens. Additionally, we have evaluated the use of these antigens (BmSERA1, BmMCFRP1 and BmPiβS1) in an enzyme-linked immunosorbent *B. microti* antibody assay (BmELISA). Our study significantly expands the pool of well-characterized *B. microti* antigens as potential diagnostic biomarkers and vaccine candidates and demonstrates their utility as screening targets in a highly sensitive and specific diagnostic antibody assay.

## Results

### Collection of sera from *Babesia microti*-infected patients and healthy control study subjects

Sera were obtained from 28 babesiosis patients from Nantucket, MA, an island that is highly endemic for babesiosis. Each patient had symptoms consistent with babesiosis*. B. microti* infection was confirmed in patients by thin blood smear and/or *B. microti* PCR testing. Sera were collected one to three weeks after the onset of symptoms in 21 patients and four to nine weeks in seven patients. Negative control sera were obtained from 23 healthy study subjects who were enrolled in a serosurvey on Block Island, RI and tested negative for *B. microti* antibody.

### Genome-wide immunoscreening of *B. microti* cDNA phage library to detect *B. microti* antigens

To conduct a genome-wide search for immunodominant *B. microti* antigens, a cDNA library in the phage expression system was constructed to potentially represent the complete proteome expressed during the asexual blood stage of parasites isolated from infected DBA/2 mice. The foreign protein is incorporated into the virion as gIIIp fusion, which retains infectivity and displays the foreign peptide in an immunologically accessible form. The average size of the cDNA fragments fused to the gIII gene (M13 phage gene encoding gIIIp surface protein) was 400 bp (small fragments: 50–300 bp and large fragments: 300–1000 bp). Following characterization, we obtained a library size of more than 10^6^ fusion phages, each displaying a *B. microti* peptide. Given the *B. microti* genome size of ~ 6.5 Mbp and 70% of the genes interrupted by short introns of 20–25 bp, this library should theoretically represent 100% coverage of the entire open reading frame of *B. microti*. The M13 phage display library expressing the *B. microti* transcriptome was screened with a pool of seven sera obtained from babesiosis patients. Serum samples were obtained from patients between 1 and 9 weeks after the onset of symptoms, but in most cases one to three weeks after the onset of symptoms. Following two rounds of panning, a total of 960 clones were isolated and amplified via PCR, before being subjected to nucleotide sequencing. The gene sequences thus obtained were aligned to the *B. microti* genome (www.piroplasmadb.org) which led to identification of 56 *B. microti* antigens that were recognized by antibodies generated against these antigens. Of the 56 proteins, 11 were predicted to be secreted or cell-surface proteins. The annotations, protein domain(s) and the phyletic patterns of these antigens where relevant are presented in Table 1.

**Table 1:** Analysis of *Babesia microti* antigenic proteins.

GeneID*	Accession	Curated Domain Architecture	Function	Comments
BmR1_03g00785	XP_012648767.1	SIG + BAHCS1	adhesion/Cell surface/exported protein	
BmR1_04g08155	XP_012650223.1	SIG + Bm-SERA1	adhesion/Cell surface/exported protein	
BmR1_02g04285	XP_012648612.1	SIG + BmMCFRP1 /MN15/BMN1–15 Maltese cross form related protein	adhesion/Cell surface/exported protein	
BmR1_03g04855	XP_012649578.1	SIG + PiβS1 domain	adhesion/Cell surface/exported protein	
BmR1_03g00690	XP_012648749.1	SIG + EGF + EGF + EGF + EGF	adhesion/Cell surface/exported protein	
BmR1_01g03455	XP_012647758.1	AP2 DNA-binding domain	Transcription	AP2
BmR1_02g00670	XP_012647909.2	TP + peptidylprolyl isomerase	Plastid protein folding protein	peptidylprolyl isomerase with a transit peptide
BmR1_03g03490	XP_012649309.1	sGTPase	Translation	elongation factor EF-1 alpha subunit
BmR1_04g06300	XP_012649865.1	SIG	adhesion/Cell surface/exported protein	
BmR1_01g01121	XP_012647300.2	Apicomplexa specific domain (173–231)	Miscellaneous	Apicomplexan+Vitrella
BmR1_04g08775	XP_012650340.1	HSP90	Chaperone	HSP90
BmR1_02g02760	XP_012648315.1	NTN-HYDROLASE	protein degradation	20 S proteasomal Peptidase subunit
BmR1_03g00420	XP_012648695.1	Histone Fold	Chromatin	histone H2A
BmR1_01g01620	XP_012647389.1	PPR repeats	RNA binding and processing	
BmR1_04g09905	XP_012650554.2	alpha-helical protein	Miscellaneous	Apicomplexan+Vitrella-specific
BmR1_02g02985	XP_012648359.1	BLBD (Biotin Lipid Binding domain)+E3_binding+dehydrogenase	Metabolism	
BmR1_02g03700	XP_012648499.1	Acetyltransferase	Chromatin	Histone acetyltransferase 1
BmR1_04g07910	XP_012650176.1	ubiquitin+UBA	Ubiquitin	
BmR1_02g03965	XP_012648551.1	RNA-Helicase	RNA binding and processing	RNA-Helicase
BmR1_03g00020	XP_012648615.2	SIG + PißS domain	adhesion/Cell surface/exported protein	
BmR1_04g07535	XP_012650105.1	SIG + BmMCFRP /MN15/BMN1–15 Maltese cross form related protein	adhesion/Cell surface/exported protein	
BmR1_02g00205	XP_012647816.1	SIG	adhesion/Cell surface/exported protein	
BmR1_01g01845	XP_012647436.1	HAD phosphatase	Miscellaneous	Ortholog of Plasmodium HAD domain ookinete protein
BmR1_03g04120	XP_012649435.1	No Hit	Miscellaneous	
BmR1_02g02345	XP_012648236.1	Pfimp2	Miscellaneous	Ortholog of Plasmodium Pfimp2 inner membrane proteins
BmR1_03g02795	XP_012649170.1	Babesia/Theileria specific domain	Miscellaneous	
BmR1_03g02390	XP_012649089.1	Actin	Cytoskeleton	Actin
BmR1_04g05965	XP_012649799.1	Enolase_N + Enolase_C	Metabolism	Enolase
BmR1_02g01625	XP_012648094.1	GDI (Rossmann fold)	Signaling	Rab GDP dissociation inhibitor beta
BmR1_04g08040	XP_012650201.1	HSP70	Chaperone	Hsp70
BmR1_04g06288	XP_012649864.1	Nudix	Metabolism	Nucleotide NDP-X processing
BmR1_01g02215	XP_012647511.1	Pep3/Vps18/deep orange family	Golgi/Vacuolar sorting	
BmR1_01g02270	XP_012647522.1	No Hit	Miscellaneous	
BmR1_03g03070	XP_012649225.1	Ribosomal_L27A	Ribosomal Protein	large subunit ribosomal protein L27Ae
BmR1_03g02570	XP_012649124.1	SF-assemblin	Cytoskeleton	SF-assemblin/beta giardin
BmR1_03g04301	XP_012649471.1	UFD1	Ubiquitin	Ubiquitin fusion degradation protein UFD1
BmR1_02g00430	XP_012647861.1	Utp11	RNA binding and processing	UTP11 U3 small nucleolar RNA-associated protein 11
BmR1_03g02185	XP_012649047.1	BBOX	Ubiquitin	
BmR1_03g03720	XP_012649355.1	Myosin-ATPase+BZIP + BetaPropeller	Cytoskeleton	
BmR1_02g02765	XP_012648316.1	Zn-binding domain	Miscellaneous	
BmR1_04g07165	XP_012650036.1	MIT + AAA + Vps4_C	Cytoskeleton	
BmR1_01g03115	XP_012647688.1	cNMP_binding+cNMP_binding	Signaling	
BmR1_03g02831	XP_012649178.2	Protein kinase	Signaling	
BmR1_01g02860	XP_012647635.1	DDRP-beta	DNA replication/processing	DNA-directed RNA polymerase II subunit B
BmR1_03g02705	XP_012649152.1	N-OB + wHTH	DNA replication/processing	Replication A32
BmR1_03g02396	XP_012649091.1	IWS1	Chromatin	
BmR1_03g01440	XP_012648898.1	RNA-Helicase	RNA binding and processing	inactive RNA helicase
BmR1_04g05525	XP_012649711.1	Peptidase_M24 + METPEPTIDASE-HTH	Miscellaneous	Metallopeptidase
BmR1_01g01485	XP_012647367.1	RNA-Helicase	RNA binding and processing	RNA-Helicase
BmR1_03g00861	XP_012648784.1	sGTPase	Signaling	
BmR1_02g02755	XP_012648314.1	sGTPase	Signaling	
BmR1_01g01670	XP_012647399.1	SIG + Thioredoxin+Thioredoxin_6+Thioredoxin	Surface/exported protein	ER lumen disulfide bond isomerase
BmR1_03g02230	XP_012649056.1	GOLD	Golgi/Vacuolar sorting	
BmR1_04g09097	XP_012650403.1	Sec. 61_beta	secretion	
BmR1_02g03525	XP_012648466.1	TM + TM + TM + TM + TM + TM + TM + TM + TM + TM + TM	Surface/exported protein	
BmR1_04g09955	XP_012650565.1	Cpn60_TCP1	Chaperone	

*Genes are listed based on their high, medium and low immunogenic ranking shown in Fig. 2.

### Immunodominant *B. microti* antigens and their genetic variability analysis

To identify the most promising diagnostic biomarkers, 30 of the 56 *B. microti* antigens with the highest antibody reactivity in a phage-ELISA (against pool of seven babesiosis patient sera as indicated above; data not shown) were selected for further antigenic characterization. To assess the possible use of the 30 *B. microti* antigens as diagnostic biomarkers, their cDNAs were cloned with a hexa-histidine tag at the N-terminus and were expressed in *E. coli*.

The expressed region for each identified antigen is shown in Supplementary Figure S1. The recombinantly expressed proteins were purified to homogeneity using affinity chromatography (data not shown). The availability of whole genome sequence of *B. microti*^22^ has made feasible *in silico* analyses of these identified immunodominant antigens. We find that the 30 most immunodominant antigens identified from the phage library immunoscreening do not show any preferential clustering and are distributed across all four chromosomes of *B. microti*. This indicates that the antigens identified are not preferentially drawn from the proteins encoded by the highly variable sub-telomeric regions^22,23^.

**Figure 1:**
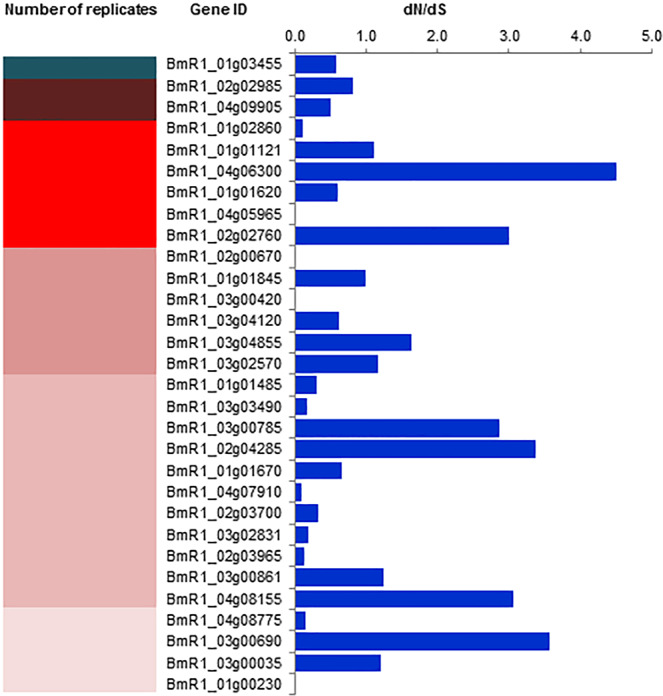
Heat map showing replicates of phages displaying 56 *B. microti* protein sequences as gIIIp fusion isolated following immunoscreening. The green color indicates the peptide domain isolated with highest frequency while other clones are represented by different intensities of red from highest to lowest frequency. The bar graph on the right shows dN/dS ratio to represent the presence of gene polymorphism as analyzed by aligning the sequences from the 41 *B. microti* isolates available on Piroplasmadb.org.

We also analyzed the genetic variability in the 30 most immunoreactive antigens by determining the non-synonymous (dN)/synonymous (dS) substitutions in relation to the recently published 41 *B. microti* genome sequences^28^. The dN/dS ratio quantifies selection pressure acting on a protein coding region. A dN/dS ratio of more than one indicates that the protein is under immune pressure and leads to changes in amino acids. In our dataset, 11 out of 30 *B. microti* proteins have dN/dS ratio of >1 suggesting selective pressure on immunodominant proteins for divergence (Fig. 1). More than half of the most immunoreactive proteins (18/30) have a dN/dS ratio of <1 suggesting a conserved nature of this subset of antigens. One of these 30 proteins has a dN/dS ratio of exactly one, raising the possibility that this gene might be under relaxed selection.

### The sensitivity of *B. microti* recombinant immunodominant antigens for detection of antibody

**Figure 2:**
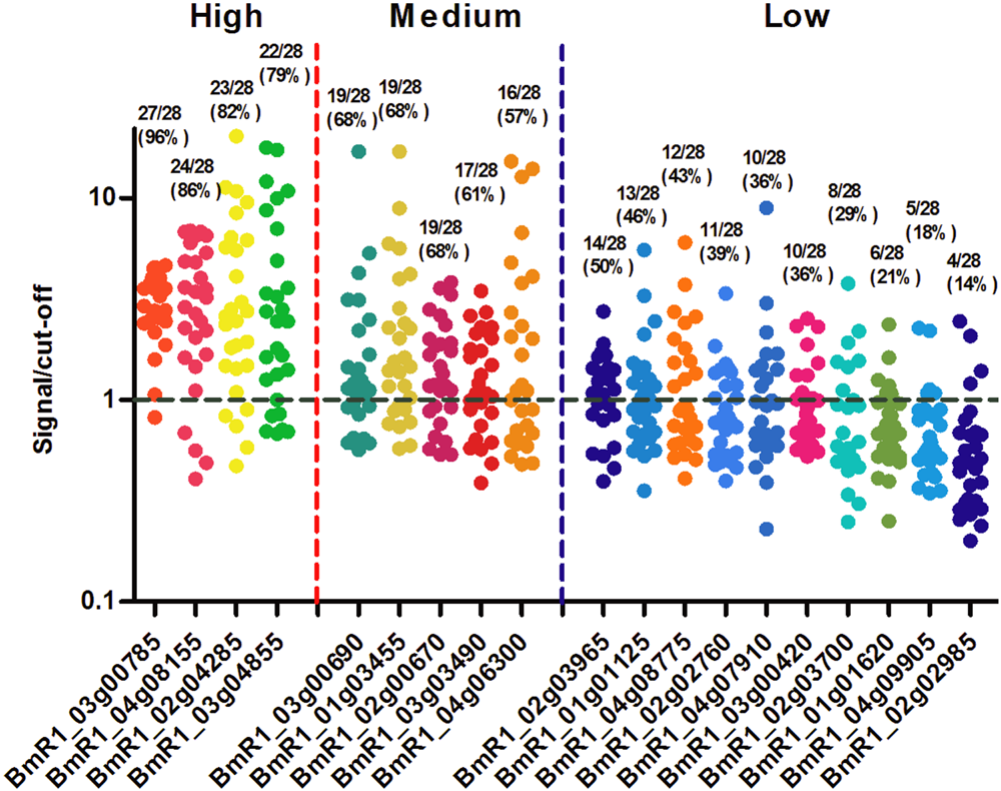
Reactivity of recombinant *B. microti* proteins against sera from *B. microti*-infected patients in BmELISA. Nineteen recombinant *B. microti* proteins were tested against 28 human sera positive for *B. microti* antibodies. Signal/cutoff is calculated by dividing the individual absorbance (A_450nm_) with the mean optical density reading for the human serum samples negative for antibodies to *B. microti* (n = 15) + 2 standard deviations of the mean. Circles represent the reactivity of each patient serum against individual recombinant protein. Immunoreactivity was classified as high, medium and low based on having the highest percentage of positives over the signal/cut-off value above 1.

The seven sera that had been used to identify antigens by phage display were screened in an ELISA that used the 30 most immunoreactive recombinant *B. microti* proteins as capture antigens. Of this screening, we further selected 19 highest immunoreactive antigens for more in- depth evaluation as capture antigen in ELISA of using sera from 28 babesiosis patients and 15 negative controls (Supplementary Table S1 and Fig. 2). The sensitivity of novel immunodominant antigens in the BmELISA varied greatly and there was a clear antigenic hierarchy. No single recombinant antigen could recognize antibody in all 28 serum samples tested. On the basis of reactivity, these 19 recombinant antigens were organized as high reactivity (reactivity against >75% of the patient sera), medium reactivity (reactivity against >50% but ≤75% of the patient sera), and low reactivity (reactivity against ≤50% of the patient sera). The three most reactive *B. microti* antigens were antigenically characterized by protein mass spectrometry and SDS-PAGE (see Supplementary text, Supplementary Figure S2 and S3).

### Babesia microti antibody ELISA (BmELISA) using three immunodominant proteins

We further determined the sensitivity of the three immunoreactive *B. microti* antigens – BmSERA1, BmMCFRP1 and BmPiβS1 individually and in a combination of three antigens in a BmELISA tested against serum samples from 28 babesiosis patients and 23 normal human serum samples. In addition, we also compared the sensitivity of these three antigens to a previously described immunodominant antigen – BmBAHCS1^26,29^. The reactivity of individual antigens in BmELISA is as follows: BmSERA1 (24/28: 86%), BmMCFRP1 (23/28: 82%), BmPiβS1 (22/28: 79%), and BmBAHCS1 (27/28: 96%). A combination of BmSERA1, BmMCFRP1 and BmPiβS1 gave a sensitivity of 96% (27/28). When BmBAHCS1 was included, the 4 antigen combination was able to recognize all 28 (100%) of the babesiosis patient samples (Table 2), thus markedly improving overall sensitivity compared to the single-antigen ELISA formats. All of the 23/23 (100%) serum samples from healthy individuals were negative in both single- or multi-antigen BmELISA format indicating a high level of specificity.

**Table 2:** BmELISA sensitivity and specificity.

Antigen*	Number of Human Serum Samples		Background
	Babesia microti Positive^#^	Normal Human Serum^@^	Signal/Cut off Ratio^&^
BmSERA1	24/28(86%)	0/23	1.01
BmMCFRP1	23/28(82%)	0/23	0.140
BmPiβS1	22/28(79%)	0/23	0.353
BmBAHCS1	27/28(96%)	0/23	0.189
BmSERA1 + BmMCFRP1 + BmPiβS1	27/28(96%)	0/23	0.350
BmSERA1 + BmMCFRP1 + BmPiβS1 + BmBAHCS1	28/28(100%)	0/23	0.245

*BmSERA1 (*Babesia microti* Serine Reactive Antigen 1), BmMCFRP1 (*Babesia microti* Maltese Cross Form Related Protein 1) BmPiβS1. (*Babesia microti* Piroplasm β-Strand Domain 1), BmBAHCS1 (*Babesia microti* Alpha Helical Cell Surface Protein 1).

^@^Normal human serum  samples.

^#^*Babesia microti* positive serum samples.

^&^Signal to cut off ratio was calculated by dividing the individual absorbance (A450) by the Mean + 2 (Standard deviation) of the 23 normal human serum samples.

### Bioinformatics analyses, annotation, and biological function of immunodominant antigens

#### Sequence analysis

We next performed a detailed sequence analysis of all 56 immunoreactive proteins identified in our study. Eleven of the 56 proteins are predicted to be cell surface or secreted proteins (Table 1). Of the 56 proteins, the seven proteins with highest immunoreactivity which is defined as having the highest percentage of positives over the signal/cut-off value above 1 (Fig. 2), six are predicted to be secreted based on sequence analysis. This suggests that our screen was successful in identifying proteins that are most likely to provoke an antibody response by virtue of being extracellularly exposed to the host immune cells. Of these 11 secreted or surface proteins five are members of protein families that have been reported as antigenic in other strains of *B. microti* or other *Babesia* species. Through a comprehensive sequence searches against the complete panel of publicly available eukaryotic genome sequences in the Genbank database we observed that a total of 11 of the immunoreactive proteins recovered in our screen showed a phyletic pattern restricted uniquely to piroplasmida or more broadly to apicomplexa and related alveolates. These include some of the proteins with highest immunoreactivity suggesting that they are likely to serve as specific diagnostic markers with no risk of cross reactivity with conserved proteins from other organisms. These findings do not discount the presence of intracellular *B. microti* antigens that are capable of eliciting an effective protective response when presented to host immune cells.

#### Annotation of the most immunodominant proteins

**Figure 3:**
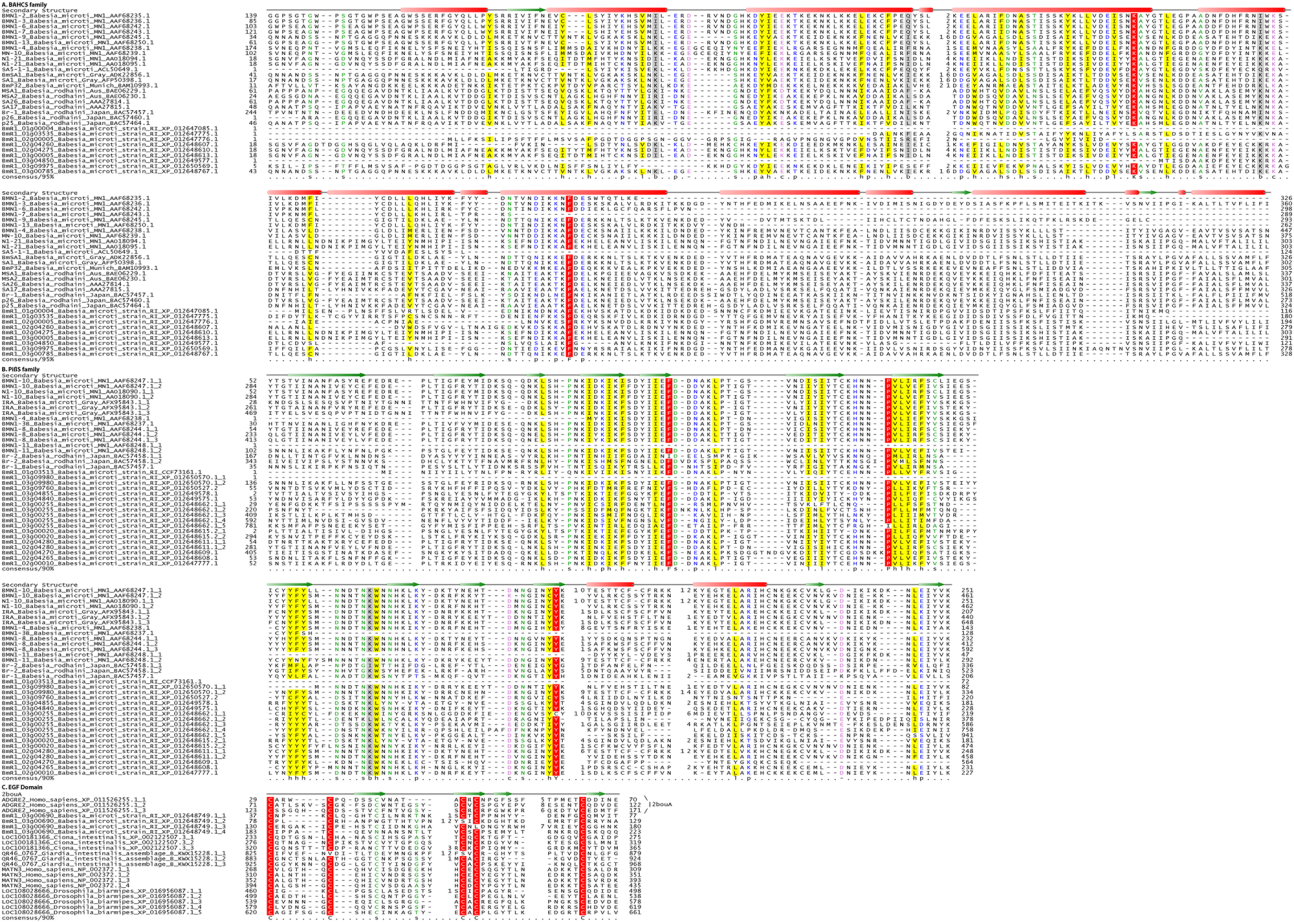
Multiple Alignment of **(A)** BAHCS family **(B)** PiβS family **(C)** EGF domain containing BmR1_03g00690 family. Representative multiple sequence alignments of the families are shown with a 95%, 90% and 90% consensus, respectively. The sequences are denoted by their gene names or gene IDs, species names, and accession numbers. Proteins with repeats are shown with underscore followed by the repeat number. The amino acid range is shown at the beginning and end of the sequence. The numbers within the alignment represent poorly conserved inserts of the given number of amino acid residues that are not shown. The predicted secondary structure or the crystal structure are shown with orange cylinders representing helices and green arrows representing beta sheets. The coloring is based on the consensus of the whole family alignment: “h” is for hydrophobic residues (ACFILMVWY [yellow]); “l” represents the aliphatic subset of the hydrophobic class [ILV (yellow]); “s” represents small residues (ACDGNPSTV (green)); “u” represents the tiny subclass of small residues (GAS [green]); “p” represents polar residues (CDEHKNQRST [blue]); “c” represents the charged subclass of polar residues (DEHKR [pink]); “—“ represents the negative subclass of charged residues (DE); “o” represents alcoholic residues (ST[orange]); and “b” represents big residues (KFILMQRWYE [gray]). Any absolutely conserved residue is labeled and shaded red.

Some of the most immunodominant proteins identified in the *B. microti* Peabody strain were cell surface proteins (BmR1_03g00690 [BmEGF1], BmR1_02g04285 [BmMCFRP1], BmR1_04g08155 [BmSERA1], BmR1_03g04855 [BmPiβS1], BmR1_03g00785 [BmBAHCS1], and BmR1_04g06300) except BmR1_01g03455 which was found to be an AP2-superfamily transcription factor typical of apicomplexa. Five of these proteins namely, BmR1_03g00690, BmR1_02g04285, BmR1_04g08155, BmR1_03g04855, and BmR1_03g00785 were subjected to an in-depth sequence analysis to obtain a better understanding of their evolutionary history and potential functional features. BmR1_03g00690 was found to contain four copies of the epidermal growth factor (EGF) domains (Fig. 3). Accordingly, we named it BmEGF1. EGF domains have been found in wide range of cytoadherence proteins from a variety of microbes in apicomplexa, such as MSP1, MSP4, MSP5, MSP8 and MSP10, which are merozoite surface proteins of *Plasmodium falciparum*^30^. The presence of EGF domains in BmR1_03g00690 was not previously known and this protein is unrelated to any EGF domain proteins from other Apicomplexa parasites, including other piroplasms. The EGF domains in this protein show certain features related to the pattern of conserved cysteines that are shared with specific EGF domains found in certain *Giardia intestinalis* and animal secreted proteins (Fig. 3). This suggests that this EGF domain protein is a unique surface protein of *B. microti*, which might have been acquired relatively recently, either from an animal source or from some other parasitic eukaryote.

The gene of a second immunodominant protein, BmR1_02g04285 (BmMCFRP1), is currently improperly predicted, where a 5′ exon containing the signal peptide has been omitted (Genbank ID: XP_021338378.1). This protein contains a conserved domain that is also found in two copies in the paralogous protein BMR1_04g07535 from *B. microti*. These two proteins in turn are homologous of the Maltese cross form-related antigen (N1–15/BMN1–15 protein; Genbank ID: BAC07532.1) that was identified in two distinct isolates (the *B. microti* Munich and MN1 strains; Table 3). They are thought to provide protection against *B. microti* infection in mice, although immunization with a control baculovirus with no *B. microti* antigen also induced some level of immunity^31,32^. Interestingly, there is extensive sequence divergence between the published Maltese cross form-related antigens from the Munich and MN1 strains and the cognate in the deposited *B. microti* R1 genome, suggesting that this protein and its paralogs might be polymorphic in *B. microti*.

**Table 3:** Selected families of *Babesia microti* surface proteins.

Family	Proteins from other strains	*Babesia microti* strain RI GeneIDs	Comments
BAHCS domain family	***B. microti*** **MN1 strain**	BmR1_01g00004	Bmn1–4 and Br-1 have both PiβS and BAHCS domains
	BMN1–2	BmR1_01g03535	
	BMN1–3	BmR1_02g00005	
	BMN1–6	BmR1_02g04260	
	BMN1–7	BmR1_02g04275	
	BMN1–9	BmR1_03g00785	
	BMN1–13	BmR1_03g04850	
	BMN1–4	BmR1_04g09975	
	MN-10	BmR1_03g00785	
	N1–21 the		
	***B. microti*** **Gray strain**: BmSA1		
	***B. microti*** **Munich strain**: BmP32		
	***B. rodhaini*** **Australia strain**: MSA1 and MSA2		
	***B. rodhaini*** **Japan strain**: Br-1, p25, and p26		
PiβS domain family	***B. microti*** **MN1 strain**	BmR1_04g09980	Divergent version in *Theileria*
	BMN1–10	BmR1_04g09760	Bmn1–4 and Br-1 has both PiβS and BAHCS domains
	N1–10	BmR1_03g04855	
	BMN1–4	BmR1_03g04840	
	BMN1–3B	BmR1_03g00255	
	BMN1–8	BmR1_03g00020	
	BMN1–11.	BmR1_02g04280	
	***B. microti*** **Gray strain**	BmR1_02g04270	
	IRA protein;	BmR1_02g04265	
	***B. rodhaini*** **Japan strain**	BmR1_02g00010	
	Br-1 and Br-2	BmR1_01g03513	
EGF domain family		BmR1_03g00690 (BMNEGF1)	Contains distinct version of EGF domain
BMNSERA family		BmR1_04g08155 (BMNSERA)	
BMN1–17/20 family	***B. microti*** **MN1 strain** BMN1–17 BMN1–20 ***B. microti*** **Gray strain**: 17N	BmR1_01g03280 (BMN1–20)	
MCFRP domain family	***B. microti*** **Munich**: MN1–15 ***B. microti*** **MN1 strain**: BMN1–15	BmR1_02g04285 (BmMCFRP1) The BmMCFRP1 gene is improperly predicted. BmR1_04g07535 (BMN1–15) is presented as fragments in Genbank in all strains except *Babesia microti* strain RI	Contains 1–2 copies of the MCFRP domain. BmR1_04g07535 additionally shares a conserved domain with BmR1_04g07660

A third immunodominant *B. microti* cell surface protein BmR1_04g08155 (BmSERA1) is a 946-amino acid protein, which was erroneously annotated as having “homologies with serine-repeat antigen 4”. This annotation is unsupported by sequence analysis and arises from improper masking of low complexity sequence. This protein has a previously described homolog in the Munich strain of *B. microti* named BmP94 and was reported to have antigenic properties consistent with those identified in the current study^33^. Remarkably, comparison of BmR1_04g08155 with this homolog protein suggests that BmR1_04g08155 is extremely fast-evolving even between these two strains, with a sequence identity of about 43%. This is much higher than the sequence divergence for other available proteins of these two strains (~95–98% identity). This divergence could be due to selection exerted by differences in the erythrocytes of potentially distinct reservoir hosts of the two strains. Alternatively, these findings might indicate that the divergence is an evolutionary response to variable immune pressure of different hosts, consistent with its character as an antigenic secreted/cell surface protein.

#### Annotation of two highly immunodominant proteins of BMN group

**Figure 4:**
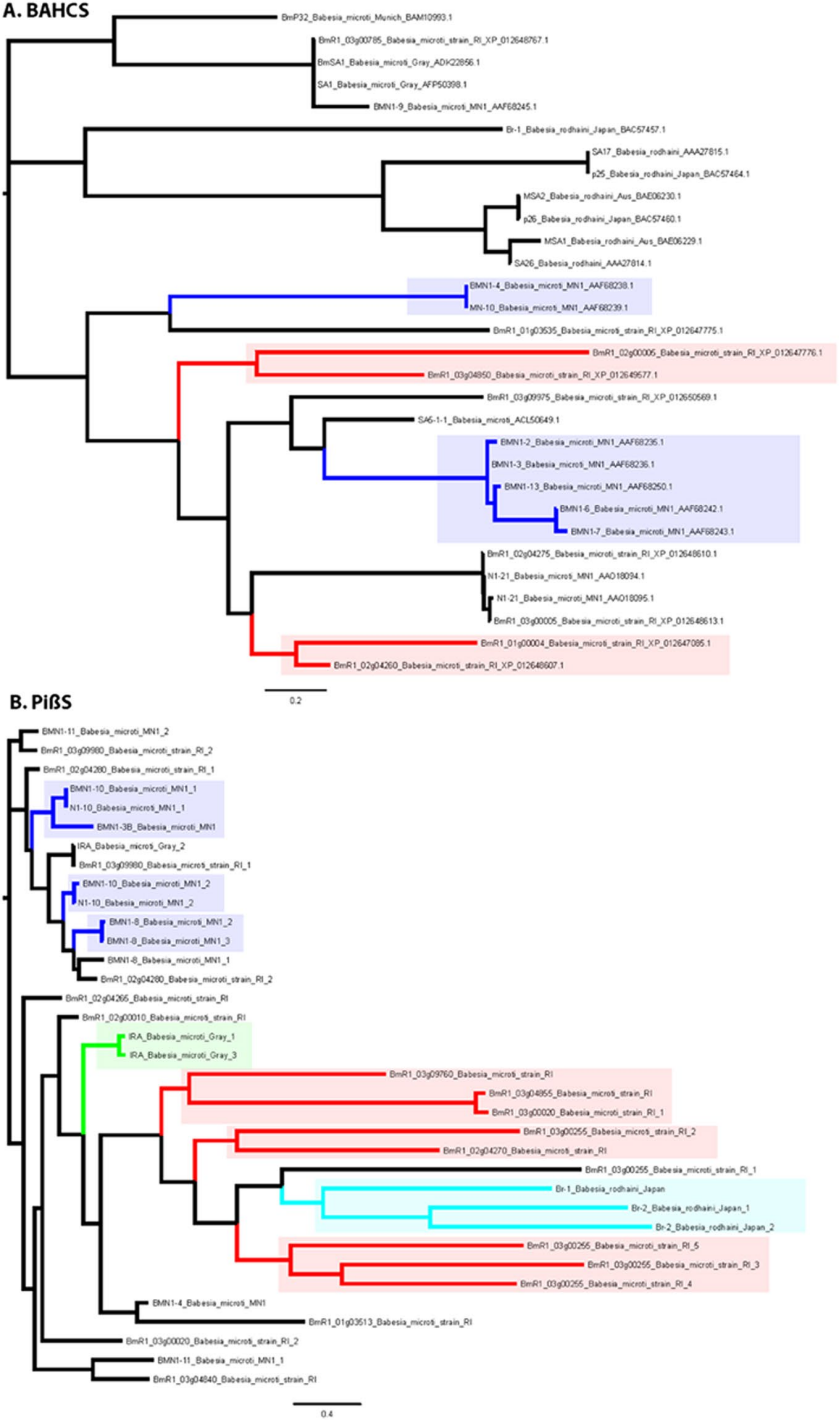
Phylogenetic tree of the **(A)** BAHCS family **(B)** BmPiβS1, and other families of proteins. Strain specific clades are shown with colored branches and boxes. *B. microti* MN1 strain is colored blue, the *B. microti* R1 strain is colored red, *B. microti* gray strain is colored green, and the *B. rodhaini* japan strain is colored light blue. The proteins are denoted by their gene names or gene ids, species names, and accession numbers.

The other two highly immunodominant proteins, BmR1_03g04855 and BmR1_03g00785 are members of the so called “BMN” class of antigenic proteins, which are shared by different *B. microti* strains and *B. rodhaini*. Some of these antigens (SA5–1–1, SA26 and SA17) were first identified in *B. rodhaini* in 1988^34^ and several subsequent studies in *B. microti* have identified them as antigenic proteins^26,31^. These studies have not fully defined the evolutionary relationships of these proteins, however, resulting in incomplete understanding of their nomenclature as reported in the literature^26,31,35,36^. Our analysis of these proteins helps to further clarify their evolutionary relationships (BmR1_03g04855 and BmR1_03g00785). Our sequence analysis shows that the proteins which have been considered BMN antigens do not constitute a monophyletic group and should not be classified as belonging to a unified “BMN family” because all members currently annotated as BMN proteins are not monophyletic. Instead our analysis shows that the majority can be classified into two largely evolutionarily unrelated groups of BMN proteins. The first of these groups includes the previously characterized BMN1–10, N1–10, BMN1–4, BMN1–3B, BMN1–8, and BMN1–11 proteins from the *B. microti* MN1 strain, the IRA protein from the *B. microti* Gray strain and the Br-1 and Br-2 proteins from the *B. rodhaini* Japan strain. The second major group is comprised of BMN1–2, BMN1–3, BMN1–6, BMN1–7, BMN1–9, BMN1–13, BMN1–4, MN-10, N1–21 from the *B. microti* MN1 strain, BmSA1 from the *B. microti* Gray strain, BmP32 from the *B. microti* Munich strain, MSA1 and MSA2 from *B. rodhaini* Australia strain and Br-1, p25, and p26 from the *B. rodhaini* Japan strain. The equivalent versions of these two groups from the *B. microti* R1 strain are provided in Table 3. Beyond these, the proteins BMN1–17 and BMN1–20 are paralogous proteins that are unrelated to any of the above groups. Likewise, the Maltese cross form-related antigen (BMN1–15/N1–15) and BmR1_02g04285 (BmMCFRP1) define a distinct family unrelated to the other BMN1 antigens (see above). Therefore, we recommend that the BMN antigens be treated as distinct groups as per their conserved domains and evolutionary relationships described here (Fig. 4, Table 3).

Our analysis shows that BmR1_03g04855 from the *B. microti* R1 strain belongs to the first of the major BMN groups (i.e. BMN1–10 and its relatives). *B. microti* R1 has a total of 10 members of this group (Table 3). These correspond in large part to the *B. microti* proteins termed BMN1 in the work by Silva *et al*.^37^. Analysis of these proteins shows that they are characterized by the presence of a conserved domain which might be present in one to five copies per protein, with a single copy in BmR1_03g04855. Secondary-structure-prediction based on the alignment of this domain showed that it contains an N-terminal region with 8 conserved β-strands followed by a C-terminal region with multiple cysteines. The N-terminal region is likely to adopt a β-sandwich fold, whereas the C-terminal region is likely to adopt a disulfide bond-supported structure. Interestingly, iterative sequence profile analysis identified proteins with a divergent version of this domain outside of *Babesia* in a group of secreted proteins in *Theileria*. While this family is expanded across *Theileria*, it is particularly abundant in the horse-parasitic species *T. equi* (~460 members); however, it is present in fewer numbers in *T. annulata*, *T. orientalis*, and *T. parva*. Because it is present in both the piroplasms, we accordingly named this domain the piroplasm β-strand (PiβS) domain (Fig. 3, Supplementary Fig. S4). Accordingly, we named BmR1_03g04855 as BmPiβS1. Given that the PiβS family is inferred to have been ancestrally present in the piroplasms, it is likely that it has played a key role in host-parasite dynamics of the entire piroplasm lineage. Importantly, our phylogenetic analysis of the PiβS domain in the genus *Babesia* shows that its evolution is dominated by lineage-specific expansions (Fig. 4). Notably, the versions in *B. rodhaini* appear to have radiated entirely independently of those from *B. microti*. Moreover, even within *B. microti* we found clades exclusively or predominantly containing R1 strain or MN1 strain proteins (Fig. 4).

Our analysis shows that BmR1_03g00785 from our screen belongs to the second major BMN group (prototyped by BMN1–2, BMN1–9 etc.). These correspond in large part to the *B. microti* proteins termed BMN2 in the work by Silva *et al*.^37^. We find that this group is characterized by a distinctive α-helical domain with 9 α-helices. Further, these proteins terminate at the C-terminus in a hydrophobic GPI-anchor sequence. This suggests that all these proteins are anchored to the cell membrane with α-helical domain lying proximal to the membrane. This domain is thus far found only in the genus *Babesia*. Nine proteins containing this domain are present in the *B. microti* R1 strain. Accordingly, we named this protein as *Babesia* α-helical cell surface (BAHCS) domain. Unlike the PiβS domain, the BAHCS domain is always found in a single copy in a protein. Thus, we named BmR1_03g00785 as BmBAHCS1. In a single protein, Br-1 from *B. rodhaini* and its cognate BMN1–4 from the *B. microti* MN1 strain, it is combined with an N-terminal PiβS domain in the same protein. Notably, phylogenetic analysis of the BAHCS domain shows a similar evolutionary trend as the PiβS domain, with independent expansions in *B. rodhaini* and *B. microti* followed by notable inter-strain radiations in the later species (Fig. 4).

In summary, our analyses suggest that the immunodominant cell surface antigens that we identified have been evolving rapidly in phylogenetically closely related organisms through independent lineage-specific expansions. Such a pattern is the hallmark of an arms race with the host and has been observed before in the case of other apicomplexan surface proteins such as the rifin-like^38^ and the var/DBL1 superfamilies in *Plasmodium falciparum*, and the vir/yir superfamilies in *P. vivax*/*P. yoelii*^30^. This suggests that the PiβS and BAHCS domain families are likely to be expressed on the cell-surface at the interface with host immune cells. The dynamic evolution suggests that the lineage-specific expansions are a positively selected response against the host immunity targeting them.

### Localization of three immunodominant proteins (BmSERA1, BmMCFRP1, and BmPiβS1) using an immunofluorescence antibody assay (IFA)

**Figure 5:**
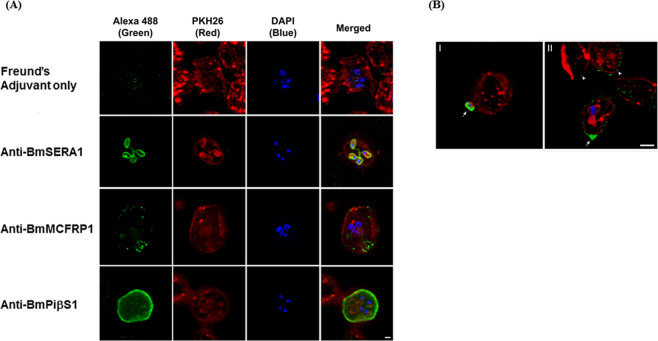
(**A**) The fluorescent image shows localization of BmSERA1, BmMCFRP1 and BmPiβS1 in *B. microti* infected mouse red blood cells (see results). Green channel (Alexa 488) images correspond to the location of *B. microti* parasite proteins. Red channel images are cell membrane stained by PKH26 and Blue channel shows DAPI stained nuclei. Merged images contain the overlay of green, red and blue channels. As observed, sera from mice immunized with Freund’s adjuvant only (negative control) shows DAPI stained nuclei demonstrating *B. microti* infected RBCs without a traceable green signal, confirming specificity of the assay. Sera collected from mice immunized with BmSERA1, BmMCFRP1 and BmPiβS1 in Freund’s Complete Adjuvant show specific localization of the *B. microti* proteins as membrane bound on the parasite surface (anti-BmSERA1) or on the surface of infected RBCs (anti-BmMCFRP1 and anti-BmPiβS1). Scale bar indicates 1 µm. (**B**) I. Free *B. microti* merozoite located near the uninfected RBC expressed BmMCFRP1 (arrow, green). Note that plasma membrane of *B. microti* and RBC were stained by PKH26 dye (red). Counterstaining by DAPI indicates *B. microti* nuclei (blue). **II**. The *B. microti* merozoite found inside RBCs showed negative staining for anti-BmMCFRP1. The apical region (entry site) of RBCs showed strong positive staining for BmMCFRP1 (arrow, green). In addition, the surface of infected and surrounding uninfected RBCs showed punctate pattern of BmMCFRP1 (arrowheads). Scale bar indicates 2 μm.

To assign a biological function to the three most immunodominant *B. microti* proteins; we investigated the localization of BmSERA1, BmMCFRP1 and BmPiβS1 proteins on intraerythrocytic *B. microti* parasites. We used immunofluorescence microscopy and polyclonal antibodies generated in mice against the respective recombinant proteins. Anti-BmSERA1 antibody showed expression of BmSERA1 on the surface of the late stage merozoite which is a tetrad structure (Fig. 5A), suggesting a possible role of this protein in invasion of *B. microti* parasites into fresh RBCs after their release from infected RBCs. The distribution of BmMCFRP1 when captured using anti-BmMCFRP1 polyclonal antibody, indicate expression on a free merozoite and on a parasite that is in the process of invading an RBC. Interestingly, we also noted a distinct scattered and punctate pattern of expression on the surface of infected RBCs. At the same time these speckled patterns are also detected in the adjacent uninfected RBCs (Fig. 5B), suggesting a possible dual function as a membrane bound and as a secreted protein that binds uninfected RBCs. The immunofluorescence signal obtained with anti-BmPiβS1 antibody shows high level expression on the surface of infected RBCs harboring tetrad form parasites and as a diffuse punctate pattern which appears to be inside the RBC cytoplasm (Fig. 5A), suggesting a role as a transported protein which is anchored in the infected RBC membrane. No non-specific reactivity was observed using antibodies against the CFA/IFA adjuvant alone formulation.

### Genetic diversity of *B. microti* immunodominant antigens (BmSERA1, BmMCFRP1, and BmPiβS1) using sequence analysis

We sought an in-depth study of the distribution and natural dynamics of the polymorphisms in the three highly reactive *B. microti* immunodominant antigens – BmSERA1, BmPiβS1 and BmMCFRP1- in endemic populations where diversity is driven by naturally acquired immunity and other biological and ecological factors. To accomplish this, we aligned the nucleotide sequences coding for the respective antigens with the sequences from the 41 *B. microti* isolates available in the Piroplasmadb.org. The full length BmSERA1, BmPiβS1 and BmMCFRP1 have a total of 72 (2841 nucleotides), 11 (816 nucleotides) and 20 (531 nucleotides) non-synonymous substitutions showing 2.5%, 1.3% and 3.7% chance of substitution, respectively. The nucleotide variation reported here as non-synonymous substitutions are mostly derived from the *B. microti* Russia-1995 strain, which is more variable at the genomic level than the strains from the continental United States^28^. When analysis for nucleotide polymorphism was limited to the continental United States isolates, the extent of non-synonymous substitutions was reduced to 23 (2841 nucleotides), 0 (816 nucleotides) and 0 (531 nucleotides) showing 0.8%, 0% and 0% chance of substitution, respectively for BmSERA1, BmPiβS1 and BmMCFRP1. We separately analyzed the amino acid sequences in BmSERA1 from the four United States isolates, which showed a minimal antigenic variation (Two from Minnesota isolates: MN-1: 1.7% and MNB010: 2.5%; one from Wisconsin: W17: 1.1%; and one from New England/North Dakota: ND-11: 0%) suggesting the diagnostic value of this molecule. Overall, our data indicate a very limited or absent genetic polymorphism among immunodominant *B. microti* antigens in the United States.

## Discussion

We utilized a phage display library encoding fragments of the *B. microti* transcriptome to identify antigenic proteins that were recognized by antibodies generated after natural infection in humans. We have identified 56 *B. microti* antigens using two cycles of affinity panning, that exhibited strong reactivity with anti-*B. microti* antibodies in patients living in a highly endemic area in the northeastern United States. We produced 30 recombinantly expressed immunoreactive *B. microti* antigens and, based on immunodominance, 19 of these were extensively screened in ELISA. Three of the most immunodominant of these antigens - BmSERA1, BmMCFRP1 and BmPiβS1 were selected for sensitivity and specificity studies in Bm-ELISA. They exhibited 86%, 82% and 79% reactivity, respectively when probed against sera from babesiosis patients. A combination of 3 antigens gave an improved sensitivity of 96% (27/28) (Table 2). Notably, in earlier studies, the BmBAHCS1 (BmR1_03g00785; named BMN1–9 by Lodes *et al*.^26^, BmSA1 by Luo *et al*.^29^, and BmGPI12 by Cornillot *et al*.^36^) allowed the detection of 100% of babesiosis serum samples tested, demonstrating the diagnostic value of this antigen. Our results clearly indicate that the discovery of these novel antigens has enhanced the repertoire  of immunodominant *B. microti* molecules that could be explored to develop highly sensitive and specific *B. microti* antibody and antigen capture assays for diagnosis of acute babesiosis, blood donor screening, and support of vaccine development. Availability of these antigens also paves the way to develop multiantigen-based *B. microti* detection assays that would offset the potential sensitivity loss due to geographical antigenic polymorphism and antigenic drift over time.

Analyses of the 30 most  highly immunoreactive *B. microti* antigens revealed that the genes encoding these proteins are distributed throughout the four *B. microti* chromosomes, suggesting a lack of clustering of genes coding for immuno-dominant antigens to a specific genomic locus. Of the 30 antigens, 11 may be subjected to immune pressure, defined by the dN/dS ratio >1 (Fig. 1). Furthermore, minimal or no genetic diversity was observed in the three most immunodominant antigens - BmSERA1, BmMCFRP1 and BmPiβS1 when sequence analyses were performed with 38 *B. microti* isolates from the continental United States^28^. These results indicate a high degree of genetic stability in *B. microti* strains circulating in the United States. They are consistent with an earlier report that *B. microti* strains in the continental United States represent minimal diversity as a geographical group, but largely differ from the published Russian strain^28^. The possible reasons for limited genetic diversity among *B. microti* isolates may include limited host specificity in invasion and survival  in red blood cells and a lack of recombination events during the sexual cycle in tick-vectors.

Further sequence and bioinformatics analyses of the five most immunodominant *B. microti* antigens - BmEGF1, BmMCFRP1, BmSERA1, BmPiβS1 and BmBAHCS1 has better revealed their domain structure, evolutionary relationships, and putative biological functions. Our sequence analysis led to a correction in annotaion of the BmSERA1 gene which was erroneously classified as homologous with serine-repeat antigen 4. A notable contribution of this study is the finding that the major group of the BMN family antigens are not monophyletic as currently annotated, but rather can be classified into two evolutionary unrelated groups of BMN proteins (see the results section). Based on our analyses, we strongly recommend that henceforth the BMN antigens be treated as distinct groups per their conserved domains and evolutionary relationships (Fig. 4, Table 3).

The localization studies by immunofluorescence microscopy further confirmed the findings of the bioinformatics analyses. The localization study revealed that BmSERA1 and BmMCFRP1 are surface exposed and secreted antigens, respectively, making them a potential immune target (Table 1). Further, BmSERA1 is distributed in a pattern that is similar to that observed in the merozoite surface protein (MSP) family in malaria parasites, *Plasmodium falciparum*^39^ and *Plasmodium vivax*^40^. MSPs in *Plasmodium* parasite have been shown to be required for the initial attachment of merozoites to RBCs for invasion. It is therefore possible that BmSERA1 may play a role in the invasion process. Given its expression pattern, BmMCFRP1 may be associated with trafficking of proteins to the surface or involved in organelle or membrane formation, as seen in *Plasmodium* and other apicomplexan parasites. In *P. falciparum*, binding of erythrocyte-binding antigen 175 (EBA-175) has been shown to prime the erythrocyte surface by altering the biophysical nature of target cell and, thereby reduces the energy barrier to invasion^41^. Thus, it is feasible that binding of BmMCFRP1 to an uninfected RBC functions in a similar fashion as *P. falciparum* EBA-175 that may serve as a gateway to invasion and new infection. Interestingly, the bright continuous fluorescent signal of BmPiβS1 is comparable to that of *Plasmodium* proteins, including knob-associated histidine-rich protein (KAHRP), ring-infected erythrocyte surface antigen (RESA), and the subtelomeric variant open reading frame protein (STEVOR)^42–45^.

In summary, our unbiased genome-wide screening for immuno-dominant *B. microti* antigens has led to the discovery of 56 novel antigens that are attractive diagnostic and vaccine targets. These antigens are available for evaluation  to develop higher sensitivity  antigen/antibody based detection assays and as vaccine candidates in experimental models of *B. microti*. Finally, our bioinformatics analyses and immunofluorescence studies allowed us to better define the structural  features and evlolutionary relationships and a finer classification of the *B. microti* BMN family antigens and assign potential biological functions to the most immunodominant *B. microti* antigens.

## Materials and methods

### *B. microti* parasites and construction of *B. microti* cDNA phage library

*B. microti* (Franca) Reichenow Peabody strain^46^ was obtained from the American Type Culture Collection (ATCC, VA). Female DBA/2NCr mice were injected with *B. microti* parasites and the parasite-infected red blood cells (RBCs) were isolated at 15–20% parasitemia. All mouse experiments were performed at White Oak animal care facility in compliance to the guidelines of White Oak Consolidated Animal Care and Use Committee (WOC ACUC). The animal study protocol used for the mouse  experiments was approved by the  WOC ACUC (protocol # 2009–03).

*B. microti* parasites were harvested by lysing the infected RBCs with Sarkosyl buffer (10 mM Tris-HCl [pH 7.5], 10 mM EDTA, 10 mM NaCl, 0.5% N-lauroylsarcosine sodium salt [Sigma-Aldrich, MO]). *B. microti* RNA was prepared using TriZol reagent (Life Technologies, NY), followed by chloroform extraction and precipitation that was performed with isopropyl alcohol and ethanol. Complimentary DNA (cDNA) encompassing the open reading frames was prepared from the *B. microti* RNA using the SMART^®^ cDNA library construction kit (Clontech laboratories Inc., CA) following manufacturer’s instructions. Briefly, the first strand cDNA was synthesized from the *B. microti* RNA using a modified oligo (dT) primer and SMART IV oligo primers. The second-strand cDNA was made using Long Distance (LD) PCR conditions. The double stranded cDNA (~10 µg) product was subjected to controlled fragmentation using sonication (Sonic Dismembrator; Thermo Fisher Scientific, MA) to generate small (50–300 bp) and large (300–1000 bp) cDNA library fragments which were separated by agarose gel electrophoresis. These cDNA fragments were dephosphorylated and polished to obtain blunt ended fragments to be ligated into *Sma* I (CCC^GGG) digested M13-derived phage vector. The ligation products were transformed into *Escherichia coli* TG1 cells (Agilent technologies, MD) and selected for recombinants (tet^r^) on tetracycline plates. Transformed cells were cultured at 37 °C with shaking at 250 rpm in 100 ml of 2XYT broth containing 5 µg/ml tetracycline for ~16 h. The recombinant lysogenic phages displaying fusion protein domains were recovered from the supernatant and the phage titer was determined. The cDNA inserts were expressed as NH_2_-terminal fusion to the gIIIp surface protein of the M13 phage. Both small (50–300 bp) and large fragment (300–1000 bp) *B. microti* libraries yielded 10^6^ independent clones, as established by limiting dilution of the transformed bacterial cells. Forty-eight clones were randomly picked from each library; PCR amplified using phage specific primers; and sequenced to determine the random distribution and diversity of the *B. microti* genome libraries.

### Genome-wide immunoscreening of *B. microti* cDNA phage library to detect *B. microti* antigens

A pool of seven babesiosis patient sera (anti-*B. microti* IFA titer >1:500) collected from babesiosis patients from Nantucket, MA were used for panning of the *B. microti* libraries. To minimize non-specific reactivity, pooled babesiosis sera were incubated on ultraviolet-killed M13K07 phage-coated petri dishes. For the affinity panning of the phage library, 96-well microwell plates (Immulon 4 HBX; Thermo Fisher Scientific, MA) were coated overnight at 4 °C with 1 µg per well of goat anti-human IgG Fcγ antibodies in phosphate-buffered saline (PBS; 10 mM Na_2_HPO_4_, 1.8 mM KH_2_PO_4_, 2.7 mM KCl, 137 mM NaCl, pH 7.4). After three washings with PBST (PBS [pH 7.4] containing 0.1% Tween 20 [Sigma-Aldrich, MO]), 5% bovine serum albumin (BSA fraction V, Sigma-Aldrich, MO) in PBST was added to the wells to block the unoccupied reactive sites. The preadsorbed babesiosis patient sera from the earlier step was added to the wells and incubated for 1 h at room temperature (RT). Wells were washed three times with PBST, and 10^8^ phages from the *B. microti* library were added for 1 h at RT. Non-adherent phages were removed by nine washes with PBST followed by three washes with PBS. The adherent phages were eluted by the addition of 0.1 N Glycine.HCl (pH 2.2), 100 µl per well for 10 min at 37 °C. The eluate was immediately neutralized by the addition of 2 M Tris (pH unadjusted). The eluate was simultaneously titrated and amplified for the next round of panning in log phase (OD_600nm_ ~0.8) *E. coli* TG1 cells. For the phage amplification, the phage infected TG1 cells were incubated at 37 °C for 90 min without shaking followed by dilution with 10 ml of 2XYT medium containing 5 µg/ml tetracycline and incubated at 37 °C, with shaking at 250 rpm for ~16 h. Phage supernatants were collected after centrifugation and one more round of panning was carried out. Phage titration plates were used for harvesting/selecting the colonies and performing PCR amplification with subsequent sequencing to establish the identity of the cloned insert. A total of 960 phage clones were sequenced using phage specific primers. The sequences obtained after Sanger’s di-deoxy sequencing (performed at the FDA center facility) were analyzed by the NCBI BLAST to identify the *B. microti* protein that each encodes. Finally, the sequencing reads were aligned to the target sequence in the MacVector program.

### Phage ELISA to select *B. microti* antigens with highest antibody affinity

The reactivity of affinity-selected phage supernatants with babesiosis patient sera was measured by ELISA. The wells of 96-well microwell plates (Immulon 4 HBX, Thermo Scientific, MA) were coated with 50 ng/well of anti-M13 phage antibody (GE Healthcare, Piscataway, NJ) and blocked with 5% skim-milk (Bio Rad, CA) PBST (0.5% Tween-20). Subsequently, phage supernatants of the selected clones were added to each well and incubated for 1 h at room temperature. Next, serially diluted sera (in 5% skim-milk PBST) were added and incubated at room temperature for 1 h. The bound antibodies were probed with horseradish peroxidase-conjugated goat anti-human IgG antibodies (Jackson Immunoresearch Laboratories, PA), and the enzymatic activity was revealed by incubating the plates with chromogenic substrate, ABTS (KPL, Inc., MD). The genes encoding the domains with high ELISA reactive phage clones were selected for cloning into an *E. coli* expression system.

### Recombinant expression and purification of *B. microti* antigens and generation of antibodies against recombinant *B. microti* antigens

Expression of recombinant protein was accomplished by amplifying either the full-length gene or a domain of it as predicted by the theoretical antigenicity index using Immune Epitope Database and Analysis Resource (IEDB, funded by National Institute of Allergy and Infectious Diseases, NIH). The putative signal and transmembrane sequences were identified using SignalP 4.1 Server and TMHMM Server v. 2.0, respectively, and excised from the constructs encompassing the protein regions selected for the recombinant expression. The PCR-amplified product was cloned into *Not*I and *Asc*I (NEB, MA) restriction site in a pET11a vector (MERCK, Germany) which was modified to include a NH2-terminal hexa-histidine tag to facilitate purification. The protein expression was carried out in *E. coli* BL-21 (λDE3) cells with the IPTG induction. Induced *E. coli* cells were lysed with the BugBuster Protein Extraction Reagent (EMD Millipore, MA) and the soluble proteins were purified from the supernatant on HisTrap column (GE Healthcare life sciences, PA). The insoluble proteins were purified by lysing the cell pellet with a combination of lysozyme and sonication, followed by buffer (50 mM Tris-HCl [pH 8.0)], 20 mM EDTA) washing 4–6 times to obtain pure inclusion bodies (IBs). The insoluble protein in the IBs was denatured in the solubilization buffer (100 mM Tris-HCl [pH 8.0], 2 mM EDTA, 6 M Guanidine HCl) before refolding under controlled redox condition in the renaturation buffer (100 mM Tris-HCl (pH 8.0), 2 mM EDTA, 500 mM L-Arginine HCl, 0.9 mM oxidized Glutathione). The refolded protein was dialyzed against a gradient of urea and finally brought into 20 mM Tris-HCl (pH 8.0) buffer and purified on HisTrap column. The purified recombinant proteins were quantified using Bradford’s reagent (Sigma-Aldrich, MO). The degree of purity of recombinant proteins was determined on SDS-PAG stained with Simply Blue Safestain. Mass-Spectrometry analysis of the purified recombinant *B. microti* proteins was performed to confirm their identity.

Female Balb/c mice (5–6 weeks old) were purchased from Jackson Laboratories (Bar Harbor, MA) and were housed at Center for Biologics Evaluation and Research (CBER) animal care facility in compliance with the guidelines of CBER Animal Care and Use Committee. Mice (5 per group) were immunized three times with 50 µg of purified recombinant BmSERA1, BmMCFRP1 and BmPiβS1 per mouse subcutaneously in Freund’s adjuvant (Complete Freund’s adjuvant (Sigma-Aldrich, MO) for the primary dose. The initial dose was followed by two booster doses in Incomplete Freund’s adjuvant) at 3-week intervals. Serum samples were collected two weeks after the third immunization and stored at −20 °C until use.

### ELISA

Individual recombinant *B. microti* antigens - BmSERA1, BmMCFRP1 and BmPiβS1 and a combination of these antigens were coated overnight (~16 h) on a 96-well Immulon 4HBX ELISA plate in PBS at 50 ng/well. Plates were washed with PBST (PBS with 0.1% Tween-20) and blocked with blocking buffer (5% skim milk PBS with 0.5% Tween-20) for 2 h at 37 °C. This was followed by washing with PBST. One hundred-fold diluted sera in blocking buffer were added to the wells and plates were incubated for 1 h at 37 °C, followed by PBST washing and then incubating with 1/10,000 diluted HRP conjugated goat anti-human IgG and IgM antibody (Jackson Immunoresearch Laboratories, PA) for an additional 1 h at 37 °C. Plates were washed six times with PBST and three times with PBS and then incubated with 50 µl per well of SureBlue Reserve TMB (KPL Inc., MD) substrate solution for an additional 10 min at room temperature. The reaction was stopped using 50 µl per well of stop solution (0.16 M H_2_SO_4_, Thermo Fisher Scientific, MA). The plates were read at 450 nm using a plate reader (SpectraMax384, Molecular devices, CA). The ELISA cutoff value was determined as follows: The mean+2 Standard Deviation (S.D.) of the negative human samples were used to determine the Cutoff Ratio. The Signal to Cutoff Ratio was then calculated by dividing the Absorbance_450_ (A_450_) of each test sample by the Cutoff Ratio. A test sample was considered as positive if the A_450_ > 1.

### Localization of three immunodominant proteins using an immunofluorescence antibody assay (IFA)

The cellular localization of native BmSERA1, BmMCFRP1, and BmPiβS1 proteins on *B. microti* parasites was determined by IFA. The *B. microti* infected RBCs grown in DBA/2 mice were washed and the RBC membrane was stained with PKH26 red fluorescent cell membrane labeling kit (Sigma-Aldrich, MO) as per manufacturer’s instructions. The stained blood cells were fixed with 3% paraformaldehyde-10 mM PIPES [piperazine-N,N’-bis (2-ethanesulfonic acid)] buffer (pH 6.4)-PBS for 30 min at room temperature, smeared on poly-L-lysine-coated glass slides, permeabilized for 10 min at room temperature with 0.25% Triton X-100-PBS, washed three times in PBS, and blocked in 5% normal goat serum (NGS)-PBS for 1 h at room temperature^47^. The slides were incubated with anti-BmSERA1, anti-BmMCFRP1 and anti-BmPiβS1 mouse serum diluted 1:100 in 2% NGS-PBS for 1 h at room temperature, washed three times in PBS, and the intra-erythrocytic parasites were counterstained for 1 h at room temperature with goat anti-mouse IgG (H + L) conjugated to Alexa Fluor 488 (Thermo Fisher Scientific, MA). The nucleus was stained with 4’, 6-diamidino-2-phenylindole (DAPI; Sigma-Aldrich, MO). The slides were washed three times with PBS in the dark, briefly swirled in distilled water, and mounted with fluoromount-G slide mounting medium (Electron microscopy sciences, VWR, PA) and sealed with a coverslip. The slides were observed with Leica TCS_SP8 DMI6000 confocal microscope system (Leica Microsystems, Germany) with a 100 × 1.4-numerical-objective (NA) oil objective lens. Images were acquired at a resolution of 1,024 by 1,024 pixels and stored in tif format for further analysis. Huygens (Scientific Volume Imaging, Hilversum, Netherlands) and Imaris (Bitplane AG, Zurich, Switzerland) software was used for deconvolution and image analysis^48^.

### Genetic diversity of *B. microti* immune-dominant antigens using sequence analysis

The presence of single nucleotide polymorphisms (SNPs) in BmSERA1 (Gene ID: BmR1_04g08155), BmPiβS1 (Gene ID: BmR1_03g04855) and BmMCFRP1 (Gene ID: BmR1_02g04285) was studied by aligning the nucleotide sequences coding for the respective antigens with the 41 *B. microti* isolates available on Piroplasmadb.org.

Protein sequence searches were performed using the iterative PSI-BLAST program against the NCBI non-redundant (NR) protein database. Multiple sequence alignments were then constructed for the individual domains using Kalign2 or Muscle, followed by manual adjustments based on profile-profile comparisons, secondary structure and structural alignments. Similarity-based clustering for both classification and culling of nearly identical sequences was performed using the BLASTCLUST program (ftp://ftp.ncbi.nih.gov/blast/documents/blastclust.html). Signal peptides and transmembrane helices were predicted using SignalP 3.0 and TMHMM v1.0 programs, respectively. Phylogenetic trees for individual families were constructed using multiple alignments of the respective domains with the FastTree program.

## Supplementary information

Supplementary Information.

## Data Availability

All data generated or analyzed during this study are included in this published article (and its Supplementary Information files).
