# Supplementary material for: Antigen Discovery, Bioinformatics and Biological Characterization of Novel Immunodominant Babesia microti Antigens
Source: Sci Rep. 2020 Jun 12;10:9598. doi: 10.1038/s41598-020-66273-6 (PMC7293334; doi:10.1038/s41598-020-66273-6)
Supplement: Supplementary file 1 — Supplementary Information. [file 41598_2020_66273_MOESM1_ESM.docx]

**Antigen Discovery, Bioinformatics and Biological Characterization of Novel Immunodominant *Babesia microti* Antigens**

**Nitin Verma, Ankit Puri, Edward Essuman, Richard Skelton, Vivek Anantharaman, Hong Zheng, Siera White, Karthigayan Gunalan, Kazuyo Takeda, Surabhi Bajpai, Timothy J. Lepore, Peter J. Krause, L. Aravind and Sanjai Kumar**

**Supplemental data**

**Supplementary Text:**

**Antigenic Characterization of BmSERA1, BmMCFRP1 and BmPiβS1.** In SDS-PAGE, recombinant BmMCFRP1 and BmPiβS1 demonstrated a gel mobility at the predicted molecular weight of ~28 kDa and ~38 kDa, respectively on SDS-PAGE, whereas, BmSERA1 showed a larger molecular mass (68 kDa) than predicted mass of 43 kDa (Supplementary Fig. S3). This anomaly is likely due to the atypical migration caused by the low-complexity QP-rich regions present in BmSERA1^1^.

1. Baylis, H. A., Allsopp, B. A., Hall, R. & Carrington, M. Characterisation of a glutamine- and proline-rich protein (QP protein) from Theileria parva. *Mol Biochem Parasitol* **61**, 171-178, doi:10.1016/0166-6851(93)90063-4 (1993).

**Supplementary Table S1.** BmELISA assay to determine the sensitivity of *B. microti* proteins.

| **Antigen** | **Number of human serum samples** | |
| --- | --- | --- |
|  | ***Babesia microti* positive** | **Normal human serum** |
| BmBAHCS1 (BmR1_03g00785) | 27/28 (96%) | 0/15 |
| BmSERA1 (BmR1_04g08155) | 24/28 (86%) | 0/15 |
| BmMCFRP1 (BmR1_02g04285) | 23/28 (82%) | 0/15 |
| BmPiβS1 (BmR1_03g04855) | 22/28 (79%) | 0/15 |
| BmEGF1 (BmR1_03g00690) | 19/28 (68%) | 0/15 |
| BmR1_01g03455 | 19/28 (68%) | 0/15 |
| BmR1_02g00670 | 19/28 (68%) | 0/15 |
| BmR1_03g03490 | 17/28 (61%) | 0/15 |
| BmR1_04g06300 | 16/28 (57%) | 0/15 |
| BmR1_02g03965 | 14/28 (50%) | 0/15 |
| BmR1_01g01125 | 13/28 (46%) | 0/15 |
| BmR1_04g08775 | 12/28 (43%) | 0/15 |
| BmR1_02g02760 | 11/28 (39%) | 0/15 |
| BmR1_04g07910 | 10/28 (36%) | 0/15 |
| BmR1_03g00420 | 10/28 (36%) | 0/15 |
| BmR1_02g03700 | 8/28 (29%) | 0/15 |
| BmR1_01g01620 | 6/28 (21%) | 0/15 |
| BmR1_04g09905 | 5/28 (18%) | 0/15 |
| BmR1_02g02985 | 4/28 (14%) | 0/15 |

**Supplementary Figure S1.** Domain architecture of *B. microti* antigens. Red bar shows the homologous domain for the specified protein; blue bar denotes the protein domain recombinantly expressed in *E. coli*; UF denotes protein with unknown function; CPP denotes conserved *Plasmodium* protein.

**
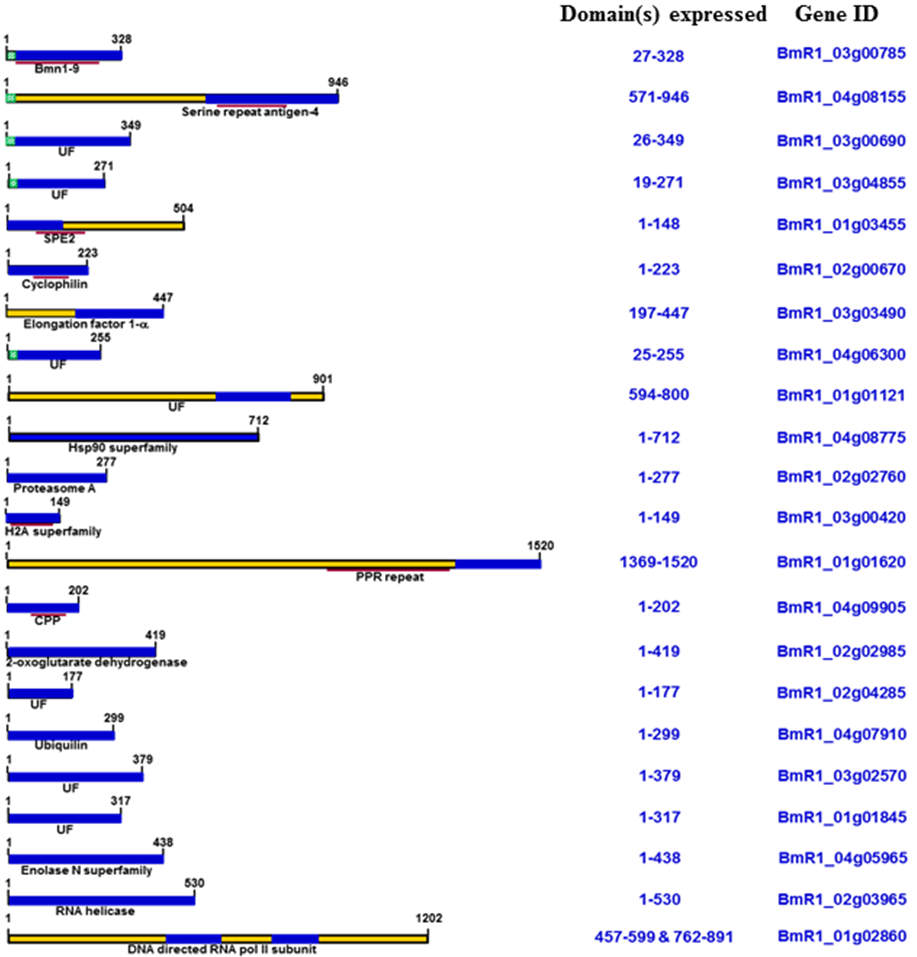
**

**Supplementary Figure S2.** Protein Mass-Spectrometry analyses of BmSERA1, BmMCFRP1, and BmPiβS1. A. BmSERA1; B. BmMCFRP1; C. BmPiβS1


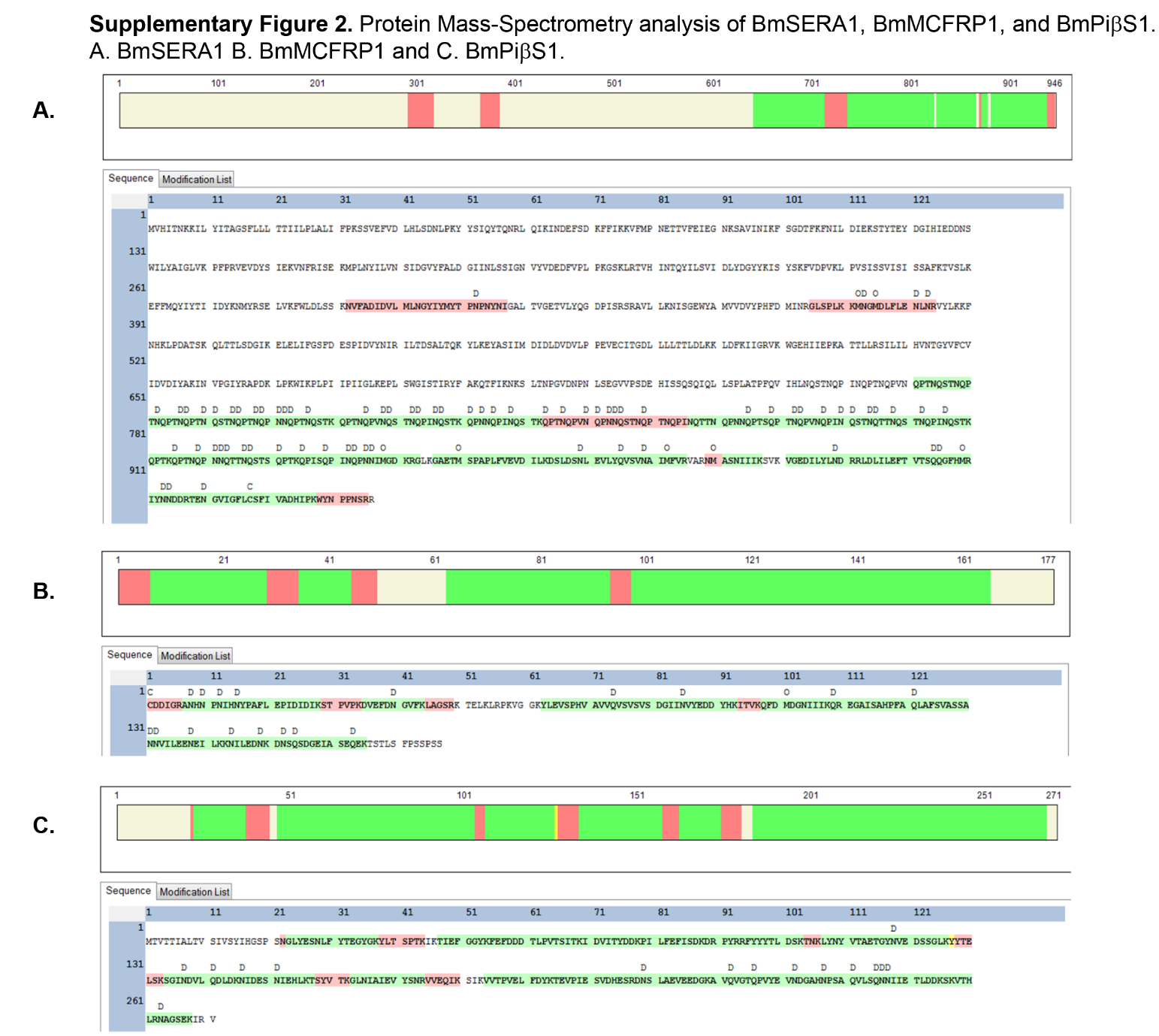


**Supplementary Figure S3.** SDS-PAGE analysis of His-tagged purified, recombinant *B. microti* proteins; BmSERA1, BmMCFRP1, and BmPiβS1. Proteins were separated on 4-12% SDS-PAGE gradient under reducing conditions and stained with Simply Blue Safestain. Lane M, Mol. Weight marker; Lane 1, BmSERA1; Lane 2, BmMCFRP1; Lane 3, BmPiβS1.

**
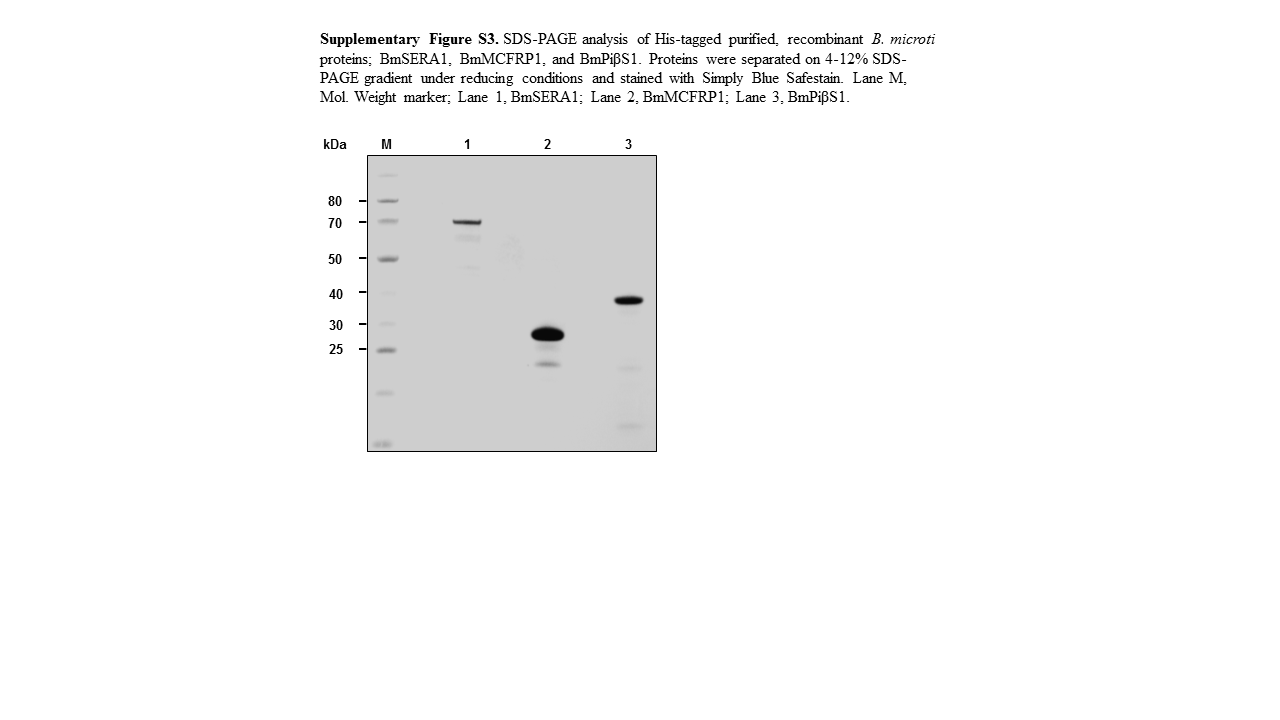
**

**Supplementary Figure S4.** Multiple Alignment of the PiβS family *Babesia* proteins with their *Theileria* orthologs.

**
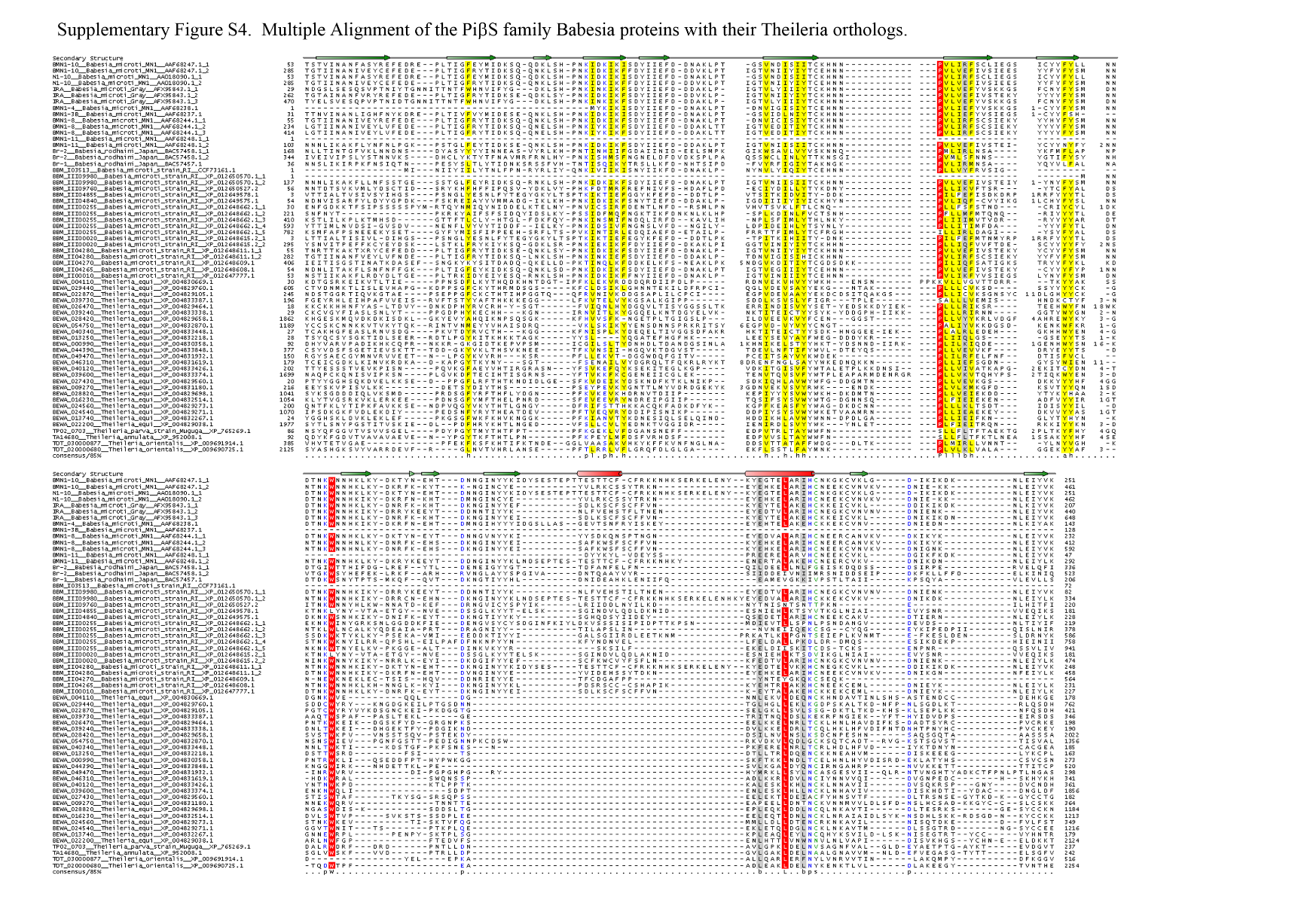
**
